# Evaluation of the performance of telephone triage service

**DOI:** 10.1186/s13049-025-01462-8

**Published:** 2025-10-22

**Authors:** Hanna Vainio, Amanda Eklund, Leena Soininen, Maaret Castrén, Paulus Torkki

**Affiliations:** 1 Department of Emergency Medicine and Services, University of Helsinki and Helsinki University Hospital, Haartmaninkatu 8, University of Helsinki, 00014 Finland; 2 Department of Industrial Engineering and Management, Department of Accounting and Business Law, Aalto University, Finland. Faculty of Medicine, University of Helsinki, Helsinki, Finland; 3 DigiFinland, Helsinki, Finland; 4 Department of Public Health, University of Helsinki, Helsinki, Finland

**Keywords:** Performance measurement, Telephone triage, Emergency medical services, Primary healthcare, Medical helpline

## Abstract

**Background:**

There is a growing need to assess the impact of the Medical Helpline (MH) 116117 on emergency care services and patient pathways. This study aims to test a validated performance measurement (PM) framework in a real-life setting and evaluate how MH influences ED service utilization and patient pathways in acute care settings.

**Methods:**

We tested the PM framework to evaluate the Finnish MH service. A quantitative before-and-after analysis was conducted using data from Vantaa for two periods: January 1 to December 31, 2016 ("Before Period") and January 1 to December 31, 2021 ("After Period"). Patient pathways were mapped by linking MH call logs and electronic health records (EHR). Data analysis included compliance and non-compliance rates for triage instructions, accessibility metrics, ED referral patterns, and cost-per-call calculations. Costs were categorized into fixed and variable components, and triage levels, diagnoses, and follow-up care outcomes were evaluated within the PM framework. The dataset included information from 146,858 patients who sought ED services during the study periods.

**Results:**

The framework provides a valuable tool for continuously measuring and improving MH performance. However, its usability highlights typical PM challenges, including data availability issues and indicator complexity. Broader validation and further development are necessary for systematic implementation. Future efforts should improve data collection processes to support more comprehensive evaluations and address identified gaps. Nevertheless, successful PM requires more than just a feasible measurement tool —it demands developing expertise in PM, refining processes, and understanding how these changes impact patient outcomes. Using a framework, PM demonstrates that the MH effectively manages high call volumes and provides timely support, particularly outside regular office hours. While patient satisfaction is generally high, advancing expertise in PM and refining processes are crucial to better understanding and enhancing patient outcomes. These efforts will further improve the quality and efficiency of MH services.

**Conclusions:**

This study addresses a critical gap in evidence-based support for assessing MH performance—an essential foundation for defining current and future care standards. The proposed framework offers actionable insights to support cost control and improve the quality of care delivery. Findings underscore the value of comprehensive monitoring across performance domains to enhance service effectiveness. Nonetheless, further refinement of the framework is necessary to more accurately capture patient outcomes and assess the real-world impact of these services. Going forward, incorporating patients’ perspectives and focusing on outcomes that are meaningful to them will be equally important. Establishing standardised performance metrics is crucial for enabling valid comparisons across different service providers.

**Trial registration:**

Not applicable.

**Supplementary Information:**

The online version contains supplementary material available at 10.1186/s13049-025-01462-8.

## Background

Performance measurement (PM) in health services has become increasingly vital due to growing demands for accountability, efficiency, and improved outcomes [1, 2]. At the same time, health systems face significant challenges, including rising costs, structural inefficiencies, and criticisms of underperformance, all of which exacerbate fluctuations in demand [3, 4]. Consequently, global priorities have emerged to evaluate care quality and implement strategies to enhance service delivery.

PM provides a structured approach to optimizing resource use, identifying inefficiencies, and linking operational processes to patient outcomes, such as waiting times, resource allocation, and compliance. By fostering continuous improvement and leveraging benchmarking to identify best practices, PM enhances health services and supports equitable care delivery aligned with strategic goals like accessibility, safety, and cost reduction [5–10].

Uniform PM frameworks are crucial for consistent evaluation and comparison across regions, but the complexity of health systems complicates their development. Addressing systemic issues requires integrated frameworks that enhance care quality and efficiency while effectively managing resources through transparent, objective performance tools [11–14].

EDs face overcrowding, leading to longer wait times, delayed treatments, and reduced care quality, particularly for critically ill patients [15, 16]. A significant factor is non-urgent visits, which strain resources and delay care for higher acuity cases [17–19]. Medical Helpline (MH) services address these challenges by guiding patients to appropriate care via phone, reducing unnecessary ED visits and improving patient flow [20–29]. Currently, there is no reliable and comprehensive framework in place to assess the overall performance of telephone triage services. In our preceding scoping review, we found that most studies evaluated the performance of telephone triage services using indicators related to health outcomes, patient experiences, or other limited perspectives [23]. This fragmentation in evaluation approaches is echoed in studies of related digital triage services, such as NHS 111 online, where Pope et al. [29] noted a lack of awareness among healthcare professionals and a crowded digital field that obscures service visibility, complicating performance assessment [30]. There was no established framework that encompassed all performance dimensions to measure overall performance.

Given the absence of a standardised or universally accepted method for assessing performance in telephone triage and related digital services, validating the indicators that will be incorporated into the framework is imperative. Beyond triage, MH services shape long-term healthcare-seeking behaviour by reducing non-urgent ED visits in regions with active systems. Their success relies on patient compliance, accurate triage, and public education. Ensuring accessibility, trust, and decision-making quality is essential for maximizing their impact and sustainability [26, 31–36].

This study aims to assess the applicability and effectiveness of a validated PM framework in a real-world setting, focusing on the Finnish MH service. The framework provides a structured approach to evaluate performance across key dimensions, such as service accessibility, efficiency, and quality. By systematically applying this framework, the study seeks to identify areas for improvement, enhance resource allocation, and demonstrate how MH services contribute to broader healthcare goals.

## Methods

### Design

This was a retrospective observational study. We tested the applicability of a previously validated PM framework in a real-life setting for the MH (see Figure S1). Additionally, we conducted a quantitative before-and-after analysis to assess how MH use influenced patient pathways and ED service utilization. The study focused on paediatric and adult patients from the city of Vantaa who sought care at joint or specialized EDs organized by Helsinki University Hospital (HUS) during two periods: January 1 to December 31, 2016 ("Before Period") and January 1 to December 31, 2021 ("After Period").

Data were retrieved from the HUS database, which aggregated EHR data and MH call logs. The dataset included 146,858 ED episodes and 25,225 MH calls. Patient pathways were mapped by linking MH call logs to subsequent ED visits using timestamps, enabling a detailed analysis of patient flow and healthcare service utilization. PIs such as ED referral frequency, adherence to self-care instructions, and hospitalization rates were compared between the two periods. Descriptive statistics were used to outline population demographics, reasons for ED visits and MH calls, and the distribution of triage categories. Finally, we compared the measurement results with previous research through a meta-analysis to identify similarities and differences in outcomes produced by the framework. This approach provided actionable insights into the effectiveness of telephone triage in managing patient flow and optimizing the use of emergency care services.

## Study setting

HUS comprises EDs for adult and paediatric patients and MH services, all available 24/7, free of charge, serving approximately 1.7 million people. Since 2018, a 24/7 MH has been available in the whole HUS area. Patients with acute conditions are advised to call MH before going to the ED after Hours, on weekends, and on public Holidays. Patients with non-urgent conditions during office hours can contact outpatient healthcare professionals at their local health centre. In an emergency, critical illness, or injury, patients should call emergency response 112. At the MH, nurses conduct telephone triage using a six-tier urgency classification protocol that follows the national principles of urgent care coordinated by the Ministry of Social Affairs and Health [37]. The nurses guide patients to the most appropriate care based on their needs or, in an emergency, forward the call to 112. Calls are documented in the EHR. There are two types of calls in MH: direct calls and callbacks. If a direct call cannot be answered immediately, callers can queue and have the option to leave a request for a callback.

### Data collection

**Fig. 1:**
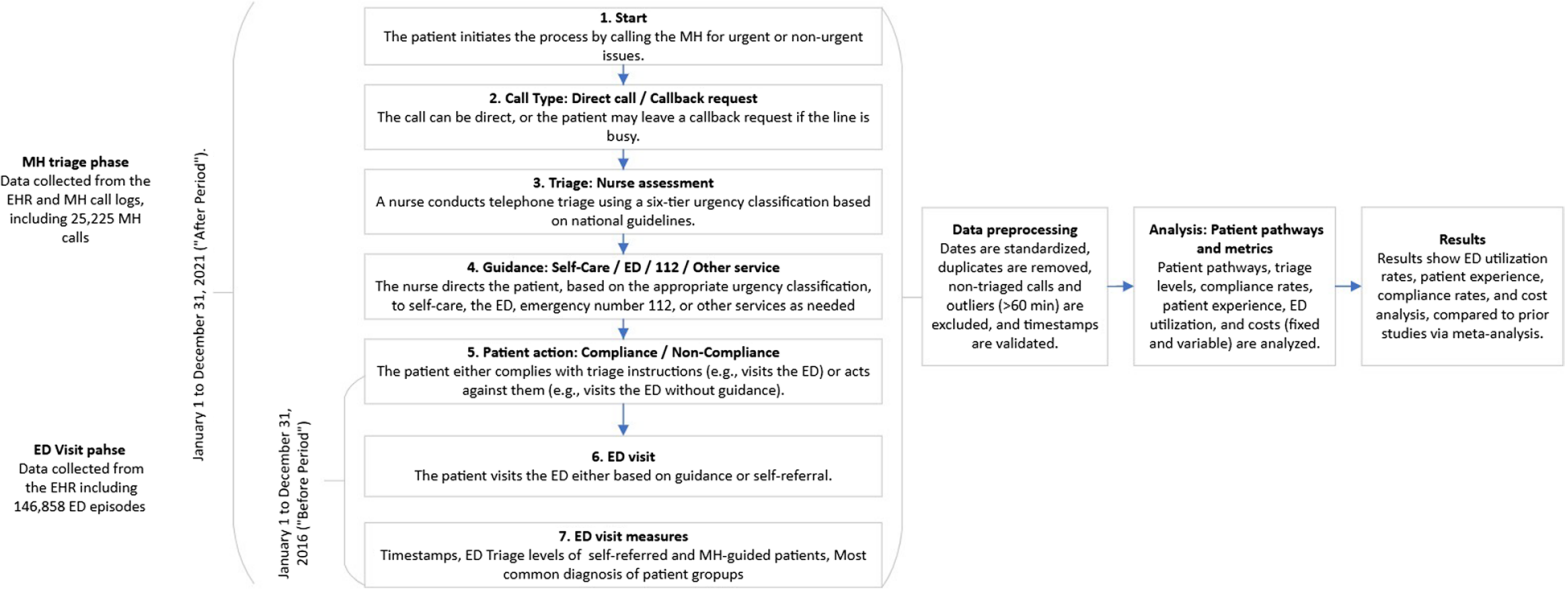
Data collection and analysis of patient pathways and the ED visit process from MH to ED

The dataset includes 146,858 patients who sought treatment at the ED during two periods: the Before Period (January 1, 2016 – December 31, 2016) and the After Period (January 1, 2021 – December 31, 2021). Data were collected from the EHR. During the Before Period, patients self-referred to the ED or arrived by ambulance and were triaged to office-hour services or registered as ED patients. In the After Period, data includes MH calls and subsequent ED visits, focusing on patients from Vantaa who visited the ED (*N* = 132,314) and/or called the MH (*N* = 25,225). Data preprocessing ensured quality and consistency. Dates were standardized, duplicate records were removed, and non-triaged calls, including outliers over 60 min, were excluded. Timestamps were decomposed into day, month, and year, while treatment periods were merged to examine interactions. Diagnoses were coded for statistical analysis. MH call logs were integrated with ED data, validating timestamps to link calls with subsequent ED visits and creating a cohesive dataset that reflects the patient journey from initial contact to treatment (see Fig. 1).

### Data analysis

We assessed the frequency distributions across patient groups, incorporating a temporal dimension in our analysis. Following patient calls to MH, we computed the total duration of the triage process. The interval between the nurse accessing the patient report and the point of triage call documentation within the EHR determined the duration. Furthermore, we analysed the timing of triage calls, quantifying assessments across different times, thereby evaluating the distribution patterns of ED check-ins. We also examined the accessibility metrics of MH, including call volumes, waiting times, and response rates, based on data from direct calls and callbacks during July-December 2021.

We could not use the utility indicator from the framework as it had not been implemented. Instead, we assessed patient experience using existing MH metrics, with responses ranging from 0 (highly unlikely) to 10 (highly likely). Patient perceptions regarding the assistance they received were answered with a simple'yes'or'no'. Quality measurement was conducted through text message surveys, in which patients were asked whether they felt they received the necessary help from MH. We delineated the distribution of follow-up care recommendations issued by MH and identified the 15 most common reasons leading to ED referrals post-MH consultation or self-referral. Additionally, we categorised the triage levels of patients presenting at the ED and the diagnoses of both patient groups.

We analysed the compliance and non-compliance rates by triage outcome classes of all MH follow-up instruction groups for patients. Compliance was defined as patients following the triage instruction to seek care at the ED when recommended.

$$Overall\;compliance=\frac{Total\;registered\;in\;ED\;(Compliance\;categories)}{Total\;patients\;(Compliance\;categories)}\times100\%$$

Non-compliance refers to patients who go to the ED without guidance—the non-compliance proportion calculated from each outcome class who sought care at the ED within 24 h post-call.

$$Overall\;noncompliance=\frac{Total\;registered\;in\;ED\;(NonCompliance\;categories)}{Total\;patients\;(NonCompliance\;categories)}\times100\%$$

We categorised costs into fixed and variable costs based on the names of account groups and shared assumptions about costs. The fixed costs encompassed cost groups that do not vary in the short term with the amount of service provided, such as rent, device investments, salaries of permanent staff, and other fixed expenses. Conversely, the variable costs included expenses related to variable labour and subcontracting. We based the classification of variable costs on the premise that these costs fluctuate by service volumes—review of costs based on document analysis of financial statement 2021. In our cost analysis, we incorporated information on call volumes from the entire MH service, including calls from all regions of Uusimaa. In addition to triage calls (*N* = 120 951), the analysis also incorporated the volumes of general advice calls (*N* = 135 095) from 2021.

$$Costs\;per\;call=\frac{Total\;costs\;for\;2021}{Number\;of\;triage\;calls+Number\;of\;general\;advice\;calls}$$

## Result

Data collection and availability challenges limited the framework's applicability due to inconsistent processes across diverse healthcare systems. Consolidating data from administrative, telephone, and EHR systems proved complex, underscoring the need for streamlined integration. Missing data, accounting for 3.62% of cases, highlighted documentation and process standardization deficiencies, which must be addressed to improve data reliability. Indicators like ICPC-2 classifications and ED diagnoses were deemed irrelevant from a PM perspective, as they primarily provided classificatory rather than actionable information, adding unnecessary complexity without functional value. It is crucial to prioritize indicators that yield meaningful, actionable insights to enhance effectiveness. Future refinements should focus on enhancing the framework's usability through broader empirical validation and iterative improvements informed by real-world applications. These efforts will help ensure the framework's reliability, feasibility, and relevance to diverse healthcare settings, making it a more robust tool for performance management.

**Fig. 2:**
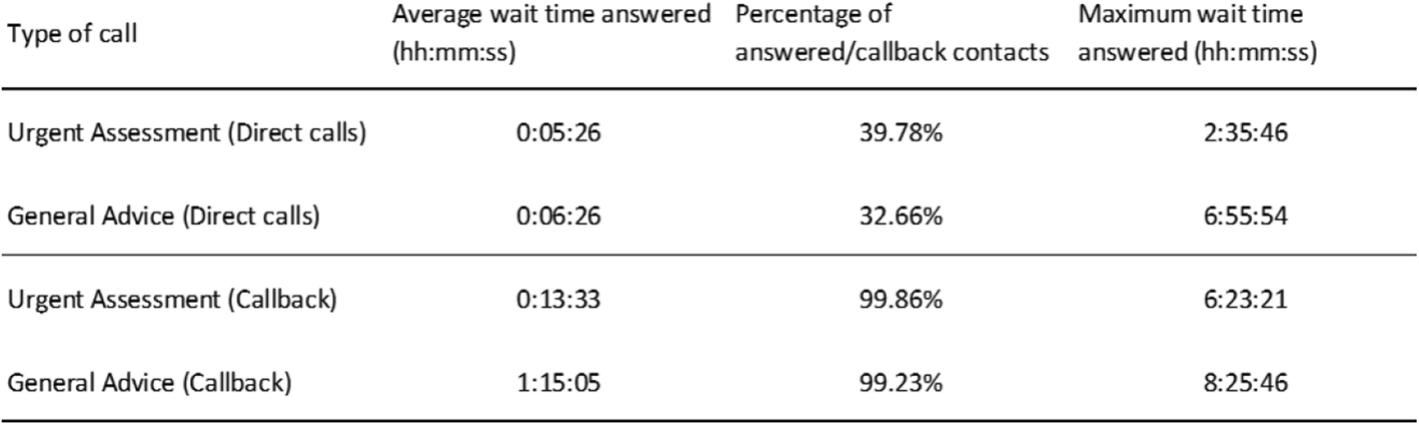
MH accessibility within the period of July to December 2021

In the MH service, 18 079 (71.76%) represented individual patients with an average age of 34. Children aged 0–12 accounted for 25.81% of the complaints. The MH service triaged 25 225 calls and processed an additional 122,908 calls, offering general health advice. Of the triaged calls, 40.25% (10 153) occurred on weekends and public Holidays and 59.75% (15 072) during weekday out-of-hours periods. The average call duration was 10 min and 23 s (Md: 8:40; Std: 6:43) (Fig. 2).

Patient experiences achieved an average of 63.4. The patients experienced receiving the necessary help from MH in 88.7% (*n* = 8 676) of contacts, while negative responses accounted for 11.2% (*n* = 1 105).

Of all triaged patients, 41.61% (*n* = 10,497) were directed to the ED. Self-care instructions were provided to 7 743 patients (30.70%), and 3 567 patients (13.4%) were referred to primary care clinics. One thousand seventy-seven patients (4.27%) were directed to take a COVID-19 test, and 602 patients (2.39%) were referred to emergency response. Written e-prescriptions were issued to 368 patients (1.46%), and additional services were offered to 272 patients (1.08%). Physician video appointments were scheduled for 187 patients (0.17%), and 912 patients (3.62%) lacked MH outcome information. The data, summarised in Table 1, delineates patient registrations, both those referred by MH and self-referred patients to ED.

**Table 1:** ED utilisation before and after MH

**Patient group**	**ED patients before MH** Before period (1.1.2016-31.12.2016)	**MH triaged patients** After period (1.1.2021-31.12.2022)	**Self-referred patients** After period (1.1.2021-31.12.2021)
Total patients	61 760 (100 %)	25 225 (100 %)	59 873 (100 %)
ED referred patients *(Compliant patients)*	N/A	5 303 (50.51 %)	N/A
ED referred patients *(Non-compliant patients)*	N/A	5 194 (49.48 %)	N/A
ED referred patients	N/A	10 497 (41.61 %)	N/A
Patients referred elsewhere (Did not visit ED)	N/A	13 097 (51.92 %)	N/A
Patients in ED from other follow-up guidance groups	N/A	1 649 (6.54 %)	N/A
Missing data	N/A	912 (3.62 %)	N/A
**ED register day and time**			
**Patient group**	**ED patients before MH** Before period (1.1.2016-31.12.2016)	**MH triaged patients** After period (1.1.2021-31.12.2022)	**Self-referred patients** After period (1.1.2021-31.12.2021)
Total patients	61 760 (100 %)	25 225 (100 %)	59 873 (100 %)
Registered at ED during office hours (Mon-Fri)	19 375 (31.37 %)	1 083 (4.29 %)	20 632 (34.5 %)
Registered at ED outside office hours (Mon-Fri)	22 767 (36.86 %)	2 549 (10.11 %)	21 507 (35.92 %)
Registered ED Monday to Friday	42 145 (68.24 %)	3 632 (14.40 %)	43 212 (72.18 %)
Registered at ED on weekends and public holidays (OOH)	19 615 (31.76 %)	3 320 (13.16 %)	16 661 (27.82 %)
Missing timestamps	3 (0.01 %)	97 (0.38 %)	1 073 (1.79 %)

The most common RFEs documented by the MH were general abdominal pain or cramps (ICPC-2 D01). At the same time, the most frequent ED diagnosis for MH-referred patients was encountered for other specified aftercare (ICD-10 Z71.8) (see Figure S2). The data highlights a significant proportion of non-specific conditions, reflecting triage outcomes complexity and alignment with subsequent ED diagnoses.

Of the triage categories (Table 2), 0.46% of self-referred patients were classified as Level 1, signifying a need for immediate medical intervention, which is marginally higher than the 0.35% of MH callers triaged in the same category. More self-referred patients (8.14%) were deemed Level 2 compared to MH callers (6.50%). The most significant disparity was noted in Level 3 – Urgent cases, where 51.05% of MH callers were triaged, as opposed to 67.69% of self-referred patients. Self-referred patients had a higher proportion of Level 4 (7.41%) and Level 5 non-urgent cases (6.86%), compared to 3.78% and 1.58%, respectively, for MH callers.

**Table 2:** Triage categories of patients registered in ED

Triage category	Triage class of self-referred patients after period *N* = 59 873 (100%)	MH 116117 called ED referred patients in after period *N* = 10 497 (100%)
Patients registered in the ED with documented triage level	*n* = 58 025 (96.91%)	*n* = 6 952 (100%)
Level 1 (Immediate)	277 (0.46%)	37 (0.35%)
Level 2 (Emergency)	4 874 (8.14%)	683 (6.50%)
Level 3 (Urgent)	40 530 (67.69%)	5 359 (51.05%)
Level 4 (Semi-urgent)	4438 (7.41%)	397 (3.78%)
Level 5 (Non-urgent)	4 113 (6.87%)	166 (1.58%)
Missing	3 793 (6.33%)	310 (2.95%)

**Fig. 3:**
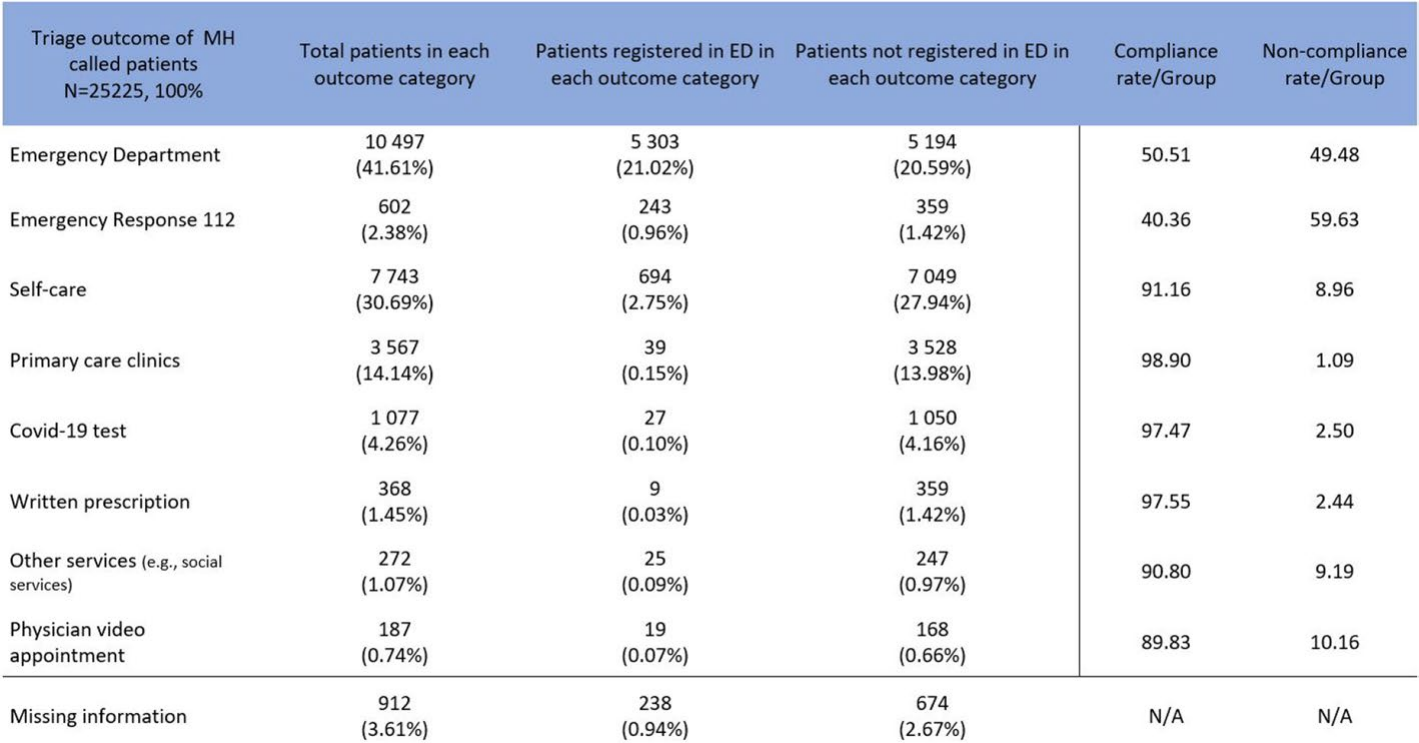
MH triage decisions and patient’s compliance rates per group

Of the triaged patients, 42.34% (*n* = 10 497) were guided in the ED (Fig. 3). The overall compliance rate, weighted by the total number of patients in each outcome category, was 71.18% (Avg. 82.07), and the overall non-compliance rate was 25.23% (Avg. 17.93). Approximately one in four patients do not follow the guidance provided by MH. The ED-referred patients group indicates that about half of the patients directed to the ED followed the recommendation.

Table 3 presents the results of PM using the framework.

**Table 3:** Results of performance measurement

Monitoring	Indicator(s)	Results	Meta-analysis	Compared to previous literature	Reference
**Performance dimension: Service Accessibility**					
Percentage of answered calls	Percentage of answered calls by line for all incoming calls	**The mean duration of triaged calls**	**The mean duration of triaged calls**	**The mean duration of triaged calls**	
		10:23 min*. Avg	11:62 min Avg	7:78 min	[69]
		*Including the call documentation in EHR	7:95 min. Pooled estimate	10:00 min	[70]
				10:23 min	[71]
				13:90 min	[72]
		Total calls: 148 133			
		The answer rate for direct calls 39.78%			
		Triaged calls *N* = 25 225 (17.12%)			
		General health advice calls *N* = 122 908 (82.97%)			
		The answer rate for direct calls 39.78%			
Waiting times	The time for an incoming call to be answered	**Triaged calls occurred weekends (**Sat-Sun and public Holidays) 40.24% (10 153)	**Weekend calls** 55.67%	**Weekends (Sat and Sun)** Inbound calls: 56.85% Outbound calls: 52.73%	[71]
		**Triaged calls occurred weekdays** (Mon to Fri) 59.75% (15 072)	**Weekday calls** 43.32%	**Weekdays (Mon to Fri)** Inbound calls: 43.14% Outbound calls: 47.26%	[71]
	The time between callback request and callback	**Average wait time answered (hh:mm** **: ** **ss):**		**Average wait time answered (hh:mm):**	
		Urgent Assessment: Direct calls 00:02:26 Call back 00:13:08		2:46 (Md.)	[73]
		General Advice: Direct calls 00:04:26 Call back 01:15:05			
**Performance dimension: Patient experience**					
The utility of the service	How does the patient rate the utility of the MH service	**Satisfaction rate**	**Satisfaction rate**	**Satisfaction rate**	
		*Did the patient receive the necessary help from MH*	*N* = 33 742	73.4% *N* = 30 402	[74]
	(Likert scale 0–10: 0–2 useless, 3–4 satisfactory, 6–8 good, 9–10 very useful)	Yes 88.7% (*n* = 8 676)	80.7% Avg	97.1% *N* = 2 486	[75]
		No 11.2% (*n* = 1 105)	75.25% Pooled estimate	72.73% *N* = 276	[76]
		NPS 63.4		80.0% *N* = 578	[77]
**Performance dimension: Quality and safety**					
Distribution of the type and grade of follow-up care	Triage outcome, percentage of patients who were guided to different grades of follow-up care	**ED referred**	**ED referred**	**ED referred**	
		41.61%	46.25%	34.2%	[75]
			Pooled estimate 66.27%	18.8%	[78]
				27.5%	[79]
				33.3%	[80]
		**Self-care**	**Self-care**	**Self-care**	
		30.69%	26.4%	43.7%	[78]
			Pooled estimate 21.95%	21.5%	[71]
				30.9%	[79]
				21.5%	[80]
		**Primary care**	**Primary care**	**Primary care**	
		14.14%	51.7%	24%	[78]
			Pooled estimate 56.01%	44.7%	[74]
				44.7%	[80]
		**Emergency response**		**Emergency response**	
		2.38%		33.7%	[71]
		Written e-prescription		Other services	
		1.45%		11%	[78]
		Other services			
		1.07%			
		Covid-19 test			
		4.26%			
		Physician video appointment			
		0.74%			
		Missing			
		3.61%			
Distribution of reasons for encounter	Distribution of the reasons for the encounter by ICPC-2 classification	R Respiratory System Issues 18.09% (*n* = 4028)	N/A	R Respiratory System Issues 13.5% (*n* = 216)	[81]
				R Respiratory System Issues 5%	[82]
		A General and Unspecified Disorders 17.01% (*n* = 3786)		A General and	[81]
				Unspecified Disorders 23.3% (*n* = 372)	
		L Musculoskeletal System Issues 16.30% (*n* = 3630)		L Musculoskeletal System Issues 21.9% (*n* = 350)	[81]
		D Digestive System Issues 14.68% (*n* = 3269)		D Digestive System Issues 18.4% (*n* = 293)	[81]
		N Neurological Issues 7.11% (*n* = 1584)		N Neurological Issues 9.1% (*n* = 145)	[81]
				N Neurological Issues 4.0%	[82]
		S Skin Problems 6.89% (*n* = 1533)		S Skin Problems N/A	[81]
		U Urological Issues 5.38% (*n* = 1197)		U Urological Issues 6.1% (*n* = 97)	[81]
		K Cardiovascular System Issues 3.90% (*n* = 869)		K Cardiovascular System Issues 15.7% (*n* = 250)	[81]
		F Eye 3.18% (*n* = 708)		F Eye 8%	[82]
**Performance dimension: The outcome of the telephone triage process**					
Patients'compliance with follow-up care instruction guidance	The proportion of patients guided to the ED by the Medical Helpline nurse and those who went to the ED	**ED ref. patients compliance**	**ED ref. patients compliance**	**ED ref. patients compliance**	
		50.51% (*n* = 5 303)	65.35% Avg	64.0%	[83]
			61.41% Pooled estimate	60.0%	[80]
				63.0%	[84]
				74.4%	[75]
		**Overall compliance/non-compliance**	**Overall compliance rate**	**Overall compliance**	
		Compliance rate of ED ref. patients	60.72 Avg	69.9%	[83]
		50.51%	68.6% Pooled estimate	62.0%	[84]
				86.8%	[75]
				55.7%	[80]
		**Non-compliance rate of ED ref patients**	**Overall non-compliance rate**		
		49.48%	39.28% Avg		
			31.4% Pooled estimate		
Review of patient medical history	The proportion of patients whose medical history was reviewed by the nurse as a part of the assessment process	Could not measure	Could not measure	Increased contact with the telephone triage service is a critical predictor of severe complications. Patients with two contacts had a 77% increased risk of severe complications. In comparison, those with three or more contacts had over four times higher risk, indicating that repeated contacts may signal worsening health conditions not recognised during initial contacts	[51]
**Performance dimension: Costs per case**					
Costs per call	The production cost of service per call	The costs per call were 10.17€ per call (triaged and general advice)	13.10 € Avg	£14.50 (€16.68)	[85]
			13.66 € Pooled estimate	£12.26 (€14.36)	[86]

## Discussion

We test the described PM framework by measuring performance in terms of availability, quality, safety, outcomes, and costs in a real-life setting for Finnish MH [23, 38]. We introduce a practical framework for the PM of MH services to provide experimental evidence of MH's performance within an ED system. This approach is justified for achieving long-term sustainability and cost-effectiveness but is also crucial from the perspective of quality [12, 39, 40].

The paper demonstrates the need to flexible and adaptable measurement framework for assessing the efficiency and quality of MH [6, 12, 39, 41–43]. Strong performance in one area can be antagonistic to another. For example, an excessive focus on efficiency can incur significant costs [12]. Alternatively, pursuing high efficiency by speeding up triage at the expense of quality can result in poorer treatment outcomes, reducing service equity. Therefore, the measurement dimensions should cover the Triple Aim or Quadruple Aim to understand the balance between various measures [11, 44].

Testing the framework with data highlighted several critical challenges, including the complexity of balancing performance dimensions and the practical interdependence of indicators. Specific indicators, such as ICPC-2 classifications and ED diagnoses, were found to be irrelevant for performance measurement, as they provided only classificatory information without actionable insights. These indicators introduced unnecessary complexity into the framework without adding functional value, underscoring the need to simplify the indicator set and focus on those that deliver meaningful and actionable results.

Data availability and fragmentation posed significant obstacles to comprehensive performance measurement, emphasising the need for refinement. The framework requires ongoing validation and adaptation to ensure that individual indicators accurately reflect efficiency, safety, and patient-centeredness. This challenge is echoed in studies of digital triage services, such as NHS 111 online, where Pope et al. [29] highlighted how low awareness among healthcare professionals, compounded by a crowded digital field and inconsistent nomenclature, obscures service visibility and complicates the evaluation of patient pathways and service performance [30]. Additionally, future assessments should evaluate how MH services meet patients'needs and expand the scope of measurement to encompass the entire patient care pathway, including post-call behaviour. This broader perspective is essential to better evaluate the trade-offs and synergies among performance dimensions and enhance the framework's overall usability.

The framework demonstrated its versatility and balance as a tool for evaluating and improving MH performance. It encompasses key dimensions such as availability, quality, safety, outcomes, and costs, integrating process, structural, and outcome indicators to enable comprehensive evaluation and targeted improvements [45]. The framework's strength lies in its development process, which prioritises selecting relevant PIs to ensure the overall relevance and utility of the framework [23]. Moreover, achieving consensus with ED professionals ensures that the framework provides appropriate and actionable information for leaders and stakeholders [38]. This approach addresses a common pitfall in performance measurement—focusing only on easily measurable or historically assessed aspects—by fostering a balanced and meaningful evaluation method. By structuring performance measurement into distinct functional groups and specific measurement sets, the framework offers a sophisticated tool to assess multiple performance dimensions and support the continuous development of MH services [45].

### Strengths and limitations of the study

To our knowledge, this study is among the first to comprehensively focus on the PM of MH services, building on our scoping review that identified gaps in existing evaluation approaches [23]. By analysing a large dataset, the study provides a detailed overview of patient urgent care pathways in Finland, both before and after the implementation of MH. The integration of MH call data into our analysis framework was particularly crucial, enabling us to investigate direct outcomes, such as reductions in unnecessary ED visits, and indirect effects, such as improved patient flow. Assessing call durations and outcomes offered valuable insights into the efficiency of MH services in addressing patient concerns and directing them to the most appropriate level of care.

However, our study has several limitations. The population was restricted to ED visits from a single city, limiting the generalizability of operational findings to other populations or regions without further validation. Additionally, we lacked data on other services patients may access post-call, such as in-hours primary care or private healthcare, which complicates the assessment of the whole patient care pathway. This challenge aligns with findings by Pope et al. [29], who noted that the invisibility of NHS 111 online, driven by a crowded digital field, inconsistent nomenclature, and poor system interoperability, hinders comprehensive evaluation of patient journeys across healthcare services [30]. These issues, also relevant to telephone triage, underscore the need for integrated data systems to capture post-call behaviour and service interactions. Consequently, we cannot fully conclude How MH impacts in-hours primary care services. Our dataset, covering 2016 and 2021, was further constrained by the exclusion of data from 2017 and 2020. The 2017 data were compromised due to a restructuring of emergency services in the study area, which affected care episode documentation. In 2020, the implementation of a new patient information system and significant shifts in ED visit patterns due to the COVID-19 pandemic rendered the data unsuitable for analysis.

Comprehensive PM requires systematic documentation of processes and outcomes to identify areas for improvement in care quality and patient outcomes [46, 47]. Our study was limited to a pre-post evaluation, which restricts the depth of insights into MH's broader impacts. A more detailed understanding would require comparative settings, as supported by Pope et al.'s [29] findings on the challenges of evaluating fragmented triage systems [30]. Future studies utilising standardised PM across diverse contexts will enable real-world evidence through benchmark-controlled settings. Benchmarking performance between providers is crucial for adopting best practices and improving operations, providing valuable data to support decision-making for clinicians and healthcare administration [46].

### Implications for clinicians and policymakers

MH services operate in a complex environment, requiring a multidimensional framework for performance measurement to equip all stakeholders with the necessary tools to improve the quality of care and clinical and administrative processes [14]. The framework's applicability extends internationally, as its focus on compliance rates (e.g., 71.18%) and resource optimisation can benefit diverse healthcare systems. However, challenges such as data fragmentation (e.g., 3.62% missing data in our study) and varying EHR availability pose barriers to implementation, particularly in countries without integrated systems. Technical requirements, including interconnected hospitals, standardised EHR platforms, and automated data collection, are essential for its success.

PM of MH services is inherently multidimensional, with each framework dimension carrying its significance. Uniform frameworks must be established, widely accepted and adapted to local and national service needs to ensure consistency and enable comparisons. Finnish-specific features, such as free ED access and a national EHR system, support the compliance rate. However, the missing data highlight documentation challenges that may not be unique to this context. Moreover, the analysis of compliance rates and resource optimisation, including patient flow management through triage, provides globally applicable insights for MH service development, transcending local constraints. Measuring accessibility, such as managing wait times to enhance patient satisfaction and reduce costs, has been proven effective with a cost per call. This approach should be more widely incorporated globally. However, their success depends on overcoming data quality challenges and integrating standardised EHR systems.

At both levels, leaders must commit to the performance management process, encompassing structural, process, and outcome measures critical to the system, organisations, and patients. We propose some methods to improve future measurements. First, harmonising MH process documentation will support ongoing benchmarking and performance evaluation, allowing for the identification of areas requiring quality improvement [48]. Second, automating data collection using different databases and EHRs will enhance the efficiency and accuracy of the analysis. There is still a need to improve the performance of service from different performance dimensions. The MH helpline effectively filters non-urgent cases and prioritises care based on severity, reducing the ED burden [22, 25, 26, 49, 50]. As shown, MH can potentially reduce unnecessary ED visits and optimise resource use, though over-triage and long wait times may offset cost savings [3, 24, 51–55]. First, effective demand management is crucial for ensuring service accessibility and reducing long waiting times, negatively impacting patient satisfaction, safety, and costs [36, 53, 56–67]. Aligning staff schedules with peak call times and improving availability during non-business hours can address these issues [25, 56, 68]. We also highlight monitoring repeated contact to improve patient safety. Repeated contact indicates worsening health conditions, highlighting the need for improved risk recognition to enhance patient safety [52]. A uniform performance measurement framework like the Triple Aim model is essential for consistent evaluation and quality improvement. For future efforts, including harmonising documentation, and automating data collection via EHR integration, we suggest considering staff well-being as an additional performance dimension [38, 48, 68]. Our framework, which incorporates perspectives from various stakeholders and is based on the Triple Aim model, has proven valuable for performance monitoring [23, 38]. The framework could be further enhanced by adding a fourth dimension focused on staff well-being [44]. Although collecting data from multiple sources may be time-consuming, the framework provides a structured, balanced, and systematic approach to internal performance management.

## Conclusion

Our study advances evidence-based support for assessing the performance of MH services, a critical prerequisite for defining current and future standards. The developed performance measurement framework proved largely feasible for evaluating MH services, providing actionable insights for cost control and improving care quality, as evidenced by a 71.18% compliance rate and 88.7% patient satisfaction. The findings confirm the importance of comprehensive, multidimensional monitoring to optimise service delivery, particularly in terms of accessibility, quality, and costs. However, the framework’s applicability is context-specific, and its generalizability is limited by our study’s focus on a single city and the lack of data on post-call service use, as similarly noted by Pope et al. [29] in the evaluation of digital triage services [30]. Further development of the framework is essential to enable more precise tracking of patient outcomes and to assess real-world effectiveness. In the future, it is crucial to emphasise patients’ perspectives and focus on outcomes that matter to them, such as satisfaction and safety. Developing standardised performance metrics is vital to enable reliable comparisons across service providers, but this requires improvements in integrated data systems and data availability.

## Supplementary Information

Additional file 1: Figure S1. Performance measurement framework.

Additional file 2: Figure S2. MH-documented reasons for ED-registered patients (ICPC-2) and corresponding ED diagnoses (ICD-10).

## Data Availability

Anonymised datasets generated during and/or analysed during the current study are available from the corresponding author upon reasonable request. Please contact: hanna.m.vainio@helsinki.fi.

## Abbreviations

ED: Emergency department
ER: Emergency response
PI: Performance indicator
PM: Performance measurement
NGT: Nominal group technique
MH: Medical Helpline 116,117
EHR: Electronic health record
NPS: Net Promoter Score
HUS: Helsinki University Hospital
SD: Standard deviation
Md: Median
RFE: Reason for encounter
