# Supplementary figures and images for: Evaluation of the performance of telephone triage service
Source: Scand J Trauma Resusc Emerg Med. 2025 Oct 22;33:172. doi: 10.1186/s13049-025-01462-8 (PMC12542025; doi:10.1186/s13049-025-01462-8)

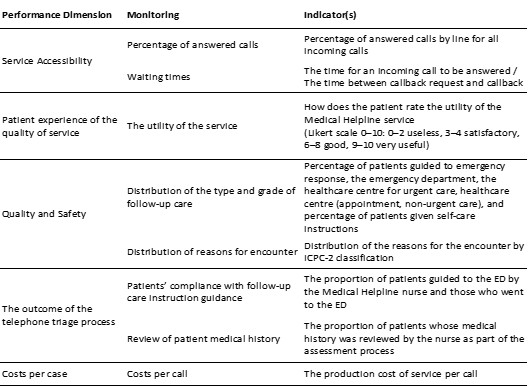

Supplement: Supplementary file 1 — Additional file 1: Figure S1. Performance measurement framework. [file 13049_2025_1462_MOESM1_ESM.jpg]

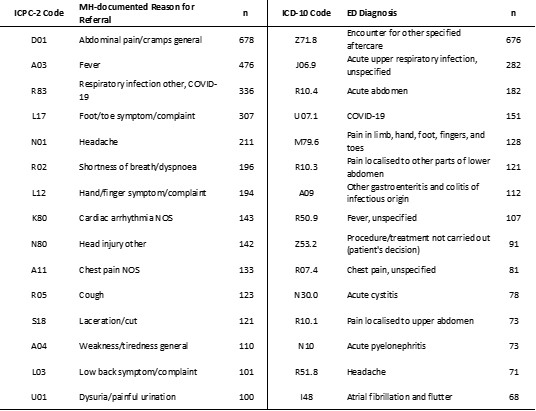

Supplement: Supplementary file 2 — Additional file 2: Figure S2. MH-documented reasons for ED-registered patients (ICPC-2) and corresponding ED diagnoses (ICD-10). [file 13049_2025_1462_MOESM2_ESM.jpg]
